# Supplementary material for: Increasing Uptake of COVID-19 Vaccination and Reducing Health Inequalities in Patients on Renal Replacement Therapy—Experience from a Single Tertiary Centre
Source: Vaccines (Basel). 2022 Jun 13;10(6):939. doi: 10.3390/vaccines10060939 (PMC9231261; doi:10.3390/vaccines10060939)
Supplement: Supplementary file 1 [file vaccines-10-00939-s001.zip › vaccines-1734515-supplementary.pdf]

<u>**Supplementary file S1**</u>

COVID VACCINE reaction guide

Dear Sir / Madam Hospital number Name

The trial data reported so far is reassuring that most patients tolerate the vaccination well. Pain at the injection site was the commonest reported side-effect. In patients who did report vaccine related local reactions, most were mild-to-moderate and resolved within 1-2 days. Patients did report increased local symptoms after the first vaccine dose. Nobody experienced a grade 4 local reaction. Most who experience side-effects find they resolve spontaneously within 2-3 days. Please grade your symptoms.

Vaccine Dose 1-Local reactions

	Mild (Grade 1)	Moderate (grade2)	Severe (Grade 3)	Potentially life threatening (Grade 4)	Most severe grade in first week Grade 0=No symptoms
Pain at injection site	Does not interfere with activity	Interferes with activity	Prevents daily activity	A&E visit or hospitalization due to pain	
Redness	2.5-5.0 cm	5-10cm	>10cm	Skin blackening or exfoliation	
Swelling	2.5-5.0 cm	5-10cm	>10cm	Skin blackening	

Vaccine Dose 1-Systemic Reactions

Fatigue and headache were reported in almost 50% of participants but severe systemic reactions occurred in less than 4%

	Mild (Grade 1)	Moderate (grade2)	Severe (Grade 3)	Potentially life threatening (Grade 4)	Most severe grade in 1st week Grade 0=No symptoms
Vomiting	1-2 times in 24 hours	>2 times in 24 hours	Requires IV hydration	A&E visit or hospitalization for hypotensive shock	
Diarrhoea	2-3 loose stool in 24 hours	4-5 loose stools in 24 hours	6 or more loose stool in 24 hours	A&E visit or hospitalization for severe diarrhoea	
Headache	Does not interfere with activity	Some interference with activity	Prevents daily routine activity	A&E visit or hospitalization for severe headache	
Fatigue/tiredness	Does not interfere with activity	Some interference with activity	Prevents daily routine activity	A&E visit or hospitalization for severe fatigue	
Chills	Does not interfere with activity	Some interference with activity	Prevents daily routine activity	A&E visit or hospitalization for severe chills	
New or worsened muscle pain	Does not interfere with activity	Some interference with activity	Prevents daily routine activity	A&E visit or hospitalization for severe new or worsened muscle pain	
New or worsened joint pain	Does not interfere with activity	Some interference with activity	Prevents daily routine activity	A&E visit or hosp. for severe new or worsened joint pain	
